# TDAG8 deficiency reduces satellite glial number and pro-inflammatory macrophage number to relieve rheumatoid arthritis disease severity and chronic pain

**DOI:** 10.1186/s12974-020-01851-z

**Published:** 2020-05-29

**Authors:** Shih-Ping Dai, Wei-Shan Hsieh, Chien-Hua Chen, Yueh-Hao Lu, Hsu-Shan Huang, Der-Ming Chang, Shir-Ly Huang, Wei-Hsin Sun

**Affiliations:** 1 Department of Life Sciences, National Central University, Jhongli, Taoyuan City, Taiwan; 2 Graduate Institute for Cancer Biology and Drug Discovery, College of Medical Science and Technology, Taipei Medical University, Taipei, Taiwan; 3 Division of Allergy, Immunology, Rheumatology, Taipei Veterans General Hospital, Taipei, Taiwan; 4 Institute of Microbiology and Immunology, National Yang-Ming University, Taipei, Taiwan; 5 Department of Life Sciences and Institute of Genome Sciences, National Yang-Ming University, Taipei, Taiwan

**Keywords:** Rheumatoid arthritis, Chronic pain, TDAG8, Satellite glial cells, Pro-inflammatory macrophages, IL-17, IL-6

## Abstract

**Background:**

The autoimmune disease rheumatoid arthritis (RA) affects approximately 1% of the global population. RA is characterized with chronic joint inflammation and often associated with chronic pain. The imbalance of pro-inflammatory and anti-inflammatory macrophages is a feature of RA progression. Glial cells affecting neuronal sensitivity at both peripheral and central levels may also be important for RA progression and associated pain. Genetic variants in the T cell death-associated gene 8 (TDAG8) locus are found to associate with spondyloarthritis. TDAG8 was also found involved in RA disease progression and associated hyperalgesia in the RA mouse model. However, its modulation in RA remains unclear.

**Methods:**

To address this question, we intra-articularly injected complete Freund’s adjuvant (CFA) into TDAG8^+/+^, TDAG8^−/−^ or wild-type mice, followed by pain behavioral tests. Joints and dorsal root ganglia were taken, sectioned, and stained with antibodies to observe the number of immune cells, macrophages, and satellite glial cells (SGCs). For compound treatments, compounds were intraperitoneally or orally administered weekly for 9 consecutive weeks after CFA injection.

**Results:**

We demonstrated that TDAG8 deletion slightly reduced RA pain in the early phase but dramatically attenuated RA progression and pain in the chronic phase (> 7 weeks). TDAG8 deletion inhibited an increase in SGC number and inhibition of SGC function attenuated chronic phase of RA pain, so TDAG8 could regulate SGC number to control chronic pain. TDAG8 deletion also reduced M1 pro-inflammatory macrophage number at 12 weeks, contributing to the attenuation of chronic RA pain. Such results were further confirmed by using salicylanilide derivatives, CCL-2d or LCC-09, to suppress TDAG8 expression and function.

**Conclusions:**

This study demonstrates that TDAG8 deletion reduced SGC and M1 macrophage number to relieve RA disease severity and associated chronic pain. M1 macrophages are critical for the development and maintenance of RA disease and pain, but glial activation is also required for the chronic phase of RA pain.

## Introduction

Rheumatoid arthritis (RA) is an autoimmune disease and characterized with chronic joint inflammation. Chronic joint inflammation causes cartilage damage and ultimately total joint destruction but is also associated with ongoing pain and increased pain [1–3]. The magnitude of pain may not necessarily be associated with the severity of the underlying disease, and pain may persist even when disease exacerbations have apparently lessened. In RA, the neurophysiological mechanisms underlying pain remain unclear. Experimental animal models of inflammatory arthritis suggest that changes in neuronal sensitivity at both peripheral and central levels may be important [4, 5]. The causes of RA-associated pain could also differ in early and late disease stages. The acute phase of pain could be associated with acute joint inflammation, but the chronic phase could be linked to inflammatory components of neuron–immune interactions and non-inflammatory components such as neuron–glia interactions or central mechanisms [6, 7].

In RA, resident macrophages become activated in synovial tissues; they along with infiltrated macrophages secrete pro-inflammatory cytokines (e.g., tumor necrosis factor α [TNFα], interleukin 6 [IL-6]), mediators, and enzymes to regulate synovial inflammation and joint destruction [8]. Activated macrophages also produce anti-inflammatory cytokines (e.g., IL-10) to promote the resolution of inflammation and tissue repair, thus ameliorating the disease. Two different macrophage phenotypes, M1 (classically activated) and M2 (alternatively activated), are responsible for producing pro- and anti-inflammatory cytokines, respectively [9]. The imbalance between pro- and anti-inflammatory cytokines (M1/M2) could be a key mechanism of rheumatic disease progression. RA patients display a more M1 macrophage profile than do people with spondyloarthritis such as psoriatic arthritis [10] or osteoarthritis [11]. An acute hypoxia environment favors M2 macrophage polarization, but chronic hypoxia triggers M1 polarization [12].

In a monoarthritis or collagenase arthritis model, arthritic rats showed satellite glial cell (SGC) proliferation and activation in dorsal root ganglia (DRG) [13, 14]. Intrathecal injection of a glial inhibitor (fluorocitrate) blocked collagenase-induced nociception [14], which suggests that SGCs play some roles in arthritic pain. IL-17A may act on glial cells to sensitize neuron function [15]. Genome-wide association studies demonstrated that genetic variants in the T cell death-associated gene 8 (TDAG8) locus are associated with spondyloarthritis [16], and T-helper 17 (Th17) cells in spondyloarthritis patients show high expression of TDAG8 gene [17]. TDAG8 gene-deficient mice show reduced number of Th17 cells and secretion of IL-17A [18]. Consistent with these data, RA disease severity and RA-evoked pain were attenuated in TDAG8 gene-deficient mice in the RA mouse model [19]. However, how TDAG8 regulates RA and RA-evoked pain in the early and late pain phases remains unclear.

In this study, we used a previously established arthritis mouse model [19] in TDAG8 gene-deficient mice to investigate whether TDAG8-modulated chronic pain and disease severity is related to immune cells or glial cells. TDAG8 gene deficiency reduced SGC number, and SGC inhibition attenuated the chronic phase of RA pain, which suggests that TDAG8 deficiency relieved the late phase of RA pain by regulating SGCs in part. Moreover, reduced M1 macrophage number but not synovial macrophage number in TDAG8–deficient mice may explain the less attenuation of acute-phase RA pain. Consistent with the results of TDAG8 deletion, long-term suppression of TDAG8 gene expression and function by using previously developed salicylanilide derivatives [20, 21], CCL-2d, and LCC-09, reduced RA pain by modulating the number of SGCs and pro-inflammatory macrophages. Accordingly, TDAG8 gene deficiency relieved RA disease severity and pain by reducing SGC number and pro-inflammatory macrophage number.

## Materials and methods

### Agents

Complete Freund’s adjuvant (CFA) and DL-fluorocitric acid (FC) barium salt were from Sigma-Aldrich. Salicylanilide derivatives CCL-2d (3-(4-Chloro-2-fluorophenyl)-7-methoxy-2H-benzo[e][1,3]-oxazine-2,4(3H)-dione) and LCC-09 (N-(3-cyanophenyl)-20,40-difluoro-4-hydroxy-[1,10-biphenyl]-3-carboxamide) were synthesized as described [20, 21]. Tofacitinib (commercial RA drug) [22] was from Selleckchem. For animal experiments, all drugs or compounds were first solved in dimethylsulfoxide and then diluted in saline before injection.

### Arthritis mouse model

Male or female ICR mice (8–12 weeks old) were purchased from BioLASCO Taiwan (Taipei) and housed 3–4 per cage under a 12-h light/dark cycle (lights on at 7:00 am) with food and water ad libitum in a temperature- and humidity-controlled environment at National Central University. TDAG8^−/−^ and TDAG8^+/+^ mice on a B6 background were generated as described [23]. The genotyping primer sequences for TDAG8^−/−^ were 5′-gaaccattagtttggctcatgtgactg/5′-cttgtgtcatgcacaaagtagatgtcc and for TDAG8^+/+^, 5′-cgaactctagctggcttttatccaataat/5′-gaaccattagtttggctcatgtgactg. Care and use of mice conformed to the Guide for the Use of Laboratory Animals (US National Research Council) and the experimental procedures were approved by the local animal use committee (IACUC, National Central University, Taiwan).

All behavioral testing was performed between 9:00 am and 5:00 pm. Efforts were made to minimize the number of animals used and their suffering. Arthritis was induced as described [19]. Briefly, TDAG8^+/+^, TDAG8^−/−^ or wild-type (WT ICR) mice were injected with 5 μg CFA in the right ankle joint once a week for 4 weeks (CFA-ctrl). Compounds (CCL-2d [360, 3600 μg/kg], LCC-09 [39, 390 μg/kg], was intraperitoneally injected once at 4w after CFA injection, followed by mechanical tests. For long-term treatment, compounds (CCL-2d [360, 3600 μg/kg], LCC-09 [39, 390 μg/kg], 3 mg/kg tofacitinib) or saline (vehicle) was intraperitoneally or orally (using oral feeding needle, ST-F173 ψ0.9 mm × L 70 mm) administered weekly for 9 consecutive weeks after CFA injection. Some experiments were only intraperitoneally injected once with CCL-2d and LCC-09, followed by the rota-rod tests (by the Taiwan Mouse clinic, Taiwan). FC (0.01 mM) was intrathecally administered at week 3 after CFA injection. Behavioral tests for mechanical or thermal stimuli were performed before and after CFA injection. In some experiments, L4-6 DRG were excised for measuring gene expression, and joints were fixed for hematoxylin and eosin (H&E) staining or immunostaining.

### Immunohistochemistry and immunostaining

The severity of the arthritis was scored from 0 to 5 as previously described [19]. Each limb was graded and given a maximum possible score of 15; the maximum score for an animal was 60.

Histological staining was performed as previously described [19]. Briefly, at 12 weeks after CFA injection, the tibiotarsal joint was excised, fixed, decalcified, embedded in paraffin and sectioned longitudinally at 10 μm with use of a microtome, then stained with HE (by the Taiwan Mouse Clinic, Taipei). Images were observed by light microscopy (Lecia, LAS EZ). Arthritic changes were scored on a scale of 0 to 5 as previously described [19]. From each joint, 6 areas of 2 sections were used to provide a representative sample of the whole joint. Cell number counted is dependent on different type of macrophages, so cell density (cells/mm^2^) is used to present the data. Mean scores were the average of all section scores for each animal. Three animals are used for each point.

Some joint sections were stained with the antibodies for CD80 (1:250, Biorbyt, UK), CD68 (1:100, Biorbyt, UK) or CD163 (1:100, Biorbyt, UK), followed by alkaline phosphatase-conjugated goat anti-rabbit IgG (1:5000, Jackson Immunoresearch). Signals were developed by nitro-blue tetrazolium chloride and 5-bromo-4-chloro-3′-Indolyphosphate *p*-toluidine (Millipore). Immunoreactivity-positive cells were counted.

Staining of SGCs was as previously described [24]. Briefly, at 0, 1, 4, 8, and 12 weeks after CFA injection, DRG isolated from vehicle- or compound-treated mice were frozen in freezing solution and cut at 12 μm by using a cryostat (Leica microsystem 3510S, Bensheim, Germany). Sections were co-stained with the antibodies for peripherin (PERI, 1:500, Sigma) and glial fibrillary acidic protein (GFAP; 1:1000; Dako), followed by TRITC-conjugated goat–anti-mouse IgG antibody (1:250, Sigma) and FITC-conjugated goat-anti-rabbit-IgG antibody (1:250, Sigma), respectively. The digitized images were captured by using MetaVue. PERI-positive neurons surrounded by GFAP-positive SGCs in one-third or more of the PERI circumference were counted (PERI^GFAP+^) and expressed as a percentage of total PERI-IR neurons (PERI_T_) in the fields analyzed (PERI^GFAP+^/PERI_T_). Data for each treatment group were collected from 10 DRG sections. The distance between two sections at least 60 μm. Total 1000–2000 PERI-positive neurons were counted for each group.

### Assessment of arthritic pain in mice

Pain behavioural tests were as described previously [25]. Briefly, mice were tested for withdrawal thresholds to mechanical stimuli applied to the plantar aspect of the hindpaw. Mice were pre-trained for 2 h each day and for 3 days before the test. Before and after CFA injection, a series (ascending force) of von Frey fibers (Touch-Test, North Coast Medical, Morgan Hill, CA) was applied. A von Frey fiber was applied to each paw 5 times at 5-s intervals. The paw withdrawal threshold (PWT) was when paw withdrawal was observed in more than 3 of 5 applications.

For thermal nociceptive response to radiant heat applied to the plantar surface of the paw, before and after CFA injection, the plantar surface of mouse hindpaws was stimulated with a lit light bulb (30% intensity, 25 s for cut-off time). The latency to withdrawal of the paw (PWL) from radiant heat was measured. Measurements from three trials at 1-min intervals in each paw were averaged. The mean basal withdrawal latencies of 15~20 s was obtained in non-injected mice.

### Measurement of cytokine levels in serum

Mice with or without compound treatments were sacrificed at 8 or 12 weeks. Blood was collected by cardiac puncture. For serum samples, blood was left to clot for 30min at 4 °C, followed by centrifugation for 20 min at 2000×*g*. Serum was aliquoted and stored at – 80 °C. TNF-α or IL-6 levels were measured with kits from R&D systems (Minneapolis, MN, USA).

### Quantitative RT-PCR

Lumbar 1–5 (L1–5) DRG ipsilateral and contralateral to injected paws were removed at 0 or 12 weeks for RNA extraction, with DRG from 0 weeks as a control. RNA extraction was performed as described [26]. Each DRG pool of each point contained at least 10 DRG from one side of 3 mice. RNA was extracted by using the RNeasy kit (Qiagen, Valencia, CA, USA). Each gene primer (100 nM), derived cDNA, and master mix (SYBR green I and AmpliTaq Gold DNA polymerase [Applied Biosystems, Foster City, CA, USA]) were mixed for PCR reactions and product detection by using the ABI Prism 7300 system. For each assay, preparations were run in triplicate. The thermal cycling conditions were 95 °C for 10 min, followed by 40 cycles of 95 °C for 15 s, and 60 °C for 1 min. The threshold cycle (Ct) values for both targets and the internal reference (mGAPDH) were measured from the same samples, and the expression of target genes relative to that of mGAPDH was calculated by the comparative Ct method.

The primer sequences for TDAG8 (197 bp) were 5′-atagtcagcgtcccagccaac (forward)/5′-cgcttcctttgcacaaggtg (reverse) and for mGAPDH (233 bp), 5′-ggagccaaacgggtcatcatctc (forward)/ 5′-gaggggccatccacagtcttct (reverse).

### Calcium imaging

To detect TDAG8-mediated signaling, calcium imaging was performed as described [24]. Briefly, human embryonic kidney, adenovirus type 5-transformed 293 cells (HEK293T, obtained from the Bioresource Collection and Research Center of Food Industry Research and Development Institute, Taiwan) were cultured on coverslips and transfected with 1.2 μg pIRES-GFP-TDAG8. Transfected cells were pre-incubated with 2.5 μM Fura-2 acetoxymethyl ester (Fura-2-AM, Molecular Probes) for 40 min in HEPES/MES buffer (125 mM NaCl, 1 mM KCl, 5 mM CaCl2, 1 mM MgCl2, 8 mM glucose, 10 mM HEPES, and 15 mM MES, pH7.6). After washing, cells were stimulated with HEPES/MES (pH 5.5) buffer, followed by [Ca^2+^]i recording with a Leica DMI3000B fluorescence microscope and a Ca^2+^ imaging system and analyzed by using MetaFluor software. The fluorescence ratio at two excitation wavelengths (340/380 nm, Ca^2+^-bound Fura-2-AM/free Fura-2-AM) was recorded and analyzed. The pH-evoked calcium transients and number of cells responding to the indicated pH values were recorded. For compound treatments, cells were stimulated with HEPES/MES buffer (pH 5.5) containing different concentrations of compounds.

### Statistical analysis

All data are presented as mean ± SEM. One-way or two-way ANOVA with post hoc Bonferroni correction was used to compare results from multiple groups. Non-parametric Mann-Whitney *U* test was used to compare results from two groups. For SGC number analysis, the *z* test for two proportions was used to test the level of significance, with 95% confidence intervals estimated. *p* < 0.05 was considered statistically significant. A statistical power analysis was performed for sample size estimation, based on data from pilot study, comparing TDAG8^−/−^ to TDAG8^+/+^ group. The effect size (ES) for *t* test was 3.4 and for two-way ANOVA was 0.30. With an alpha = 0.05 and power = 0.80, the sample size needed with the effect size (GPower 3.1) was approximately *N* = 3/group for the t test or total *N* = 48 for two–way ANOVA. Thus, our sample size of *N* ≥ 3 or *N* ≥ 48 was adequate.

## Results

### RA mice display increased number of SGCs

**Fig. 1:**
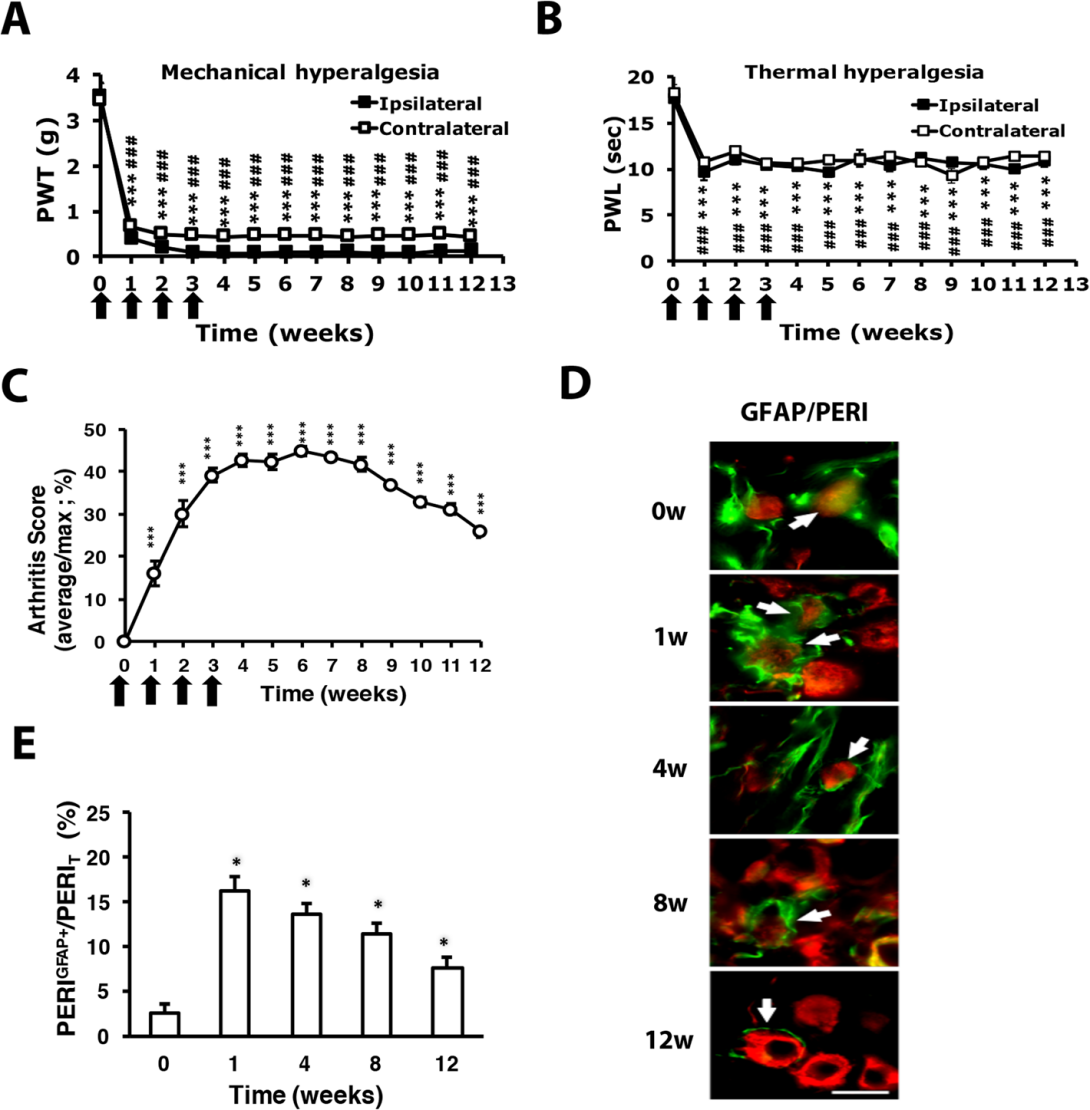
Intra-articular repeated administration of complete Freund’s adjuvant (CFA) induces long-term hyperalgesia and increases the satellite glial cell number. ICR mice (8–12 weeks old) were injected with 5 μl of 100% CFA (5 μg) in the right ankle joint (ipsilateral joint) four times at 1-week intervals, then underwent mechanical (**a**, *n* = 6) or thermal (**b**, *n* = 6) behavioral tests. Arrows are CFA treatments. Data are mean ± SEM of total tested mice. ***p < 0.001, 0 week vs other weeks on the ipsilateral side and ###*p* < 0.001, 0 week vs other weeks on the contralateral side by two-way ANOVA. PWT, paw withdrawal threshold; PWL, paw withdrawal latency. **c** Severity of arthritis presented as arthritis scores (percentage of maximum scores, *n* = 6). ***p < 0.001, 0 week vs other weeks by one-way ANOVA. **d** Dorsal root ganglia (DRG) were taken at 0, 1, 4, 8, and 12 weeks after CFA injection, then co-immunostained with anti-glial fibrillary acidic protein (GFAP) and anti-peripherin (PERI) antibodies. Cell images showing GFAP- (green) or PERI (red)-positive neurons. Scale bar is 50 μm. **e** Histograms represent the mean ± SEM percentage of PERI-positive neurons surrounded by GFAP^+^ (PERI^GFAP+^) to total PERI-positive (PERI_T_) neurons. *p < 0.05 by the *z* test

RA mice were established as previously described [19] and exhibited long-term bilateral mechanical (Fig. 1a) and thermal hyperalgesia (Fig. 1b). In the first week after CFA injection, the mean arthritis score (measuring joint, toe, and paw swelling of four limbs) was 16.1 ± 2.9% (maximum 100%). The severity of joint swelling increased with time and reached a peak at 4 weeks (42.8 ± 1.7%), remained for 8 weeks and slightly decreased at 12 weeks (25.8 ± 1.3%) (Fig. 1c). In our previous study, no synovial inflammation and swelling in saline-injected joints [19]. In RA mice, we observed swelling in uninjected joints as arthritis scores are more than one limb (each limb for 25%, arthritis scores are 42% after 4 weeks), although we did not detect dramatic synovial inflammation in uninjected joints. However, we did find bilateral hyperalgesia and raised serum cytokines TNFα, IL-7, and IL6 in arthritis mice. It suggests that polyarthritis occurred in RA mice. In a monoarthritis model or collagenase arthritis model, arthritic rats showed SGC proliferation and activation [13, 14]. To determine whether the number of SGCs was also increased in our RA mice, DRG from RA mice were immunostained for anti-GFAP (a SGC marker). Most GFAP(+) cells surrounded PERI(+) (a nociceptor marker) neurons (Fig. 1d). The number of PERI(+) neurons surrounded by GFAP(+) cells (PERI^GFAP+^) was increased and peaked in the first week, then declined slightly later (Fig. 1e).

### TDAG8 deletion reduces mechanical and thermal hyperalgesia and arthritis scores

**Fig. 2:**
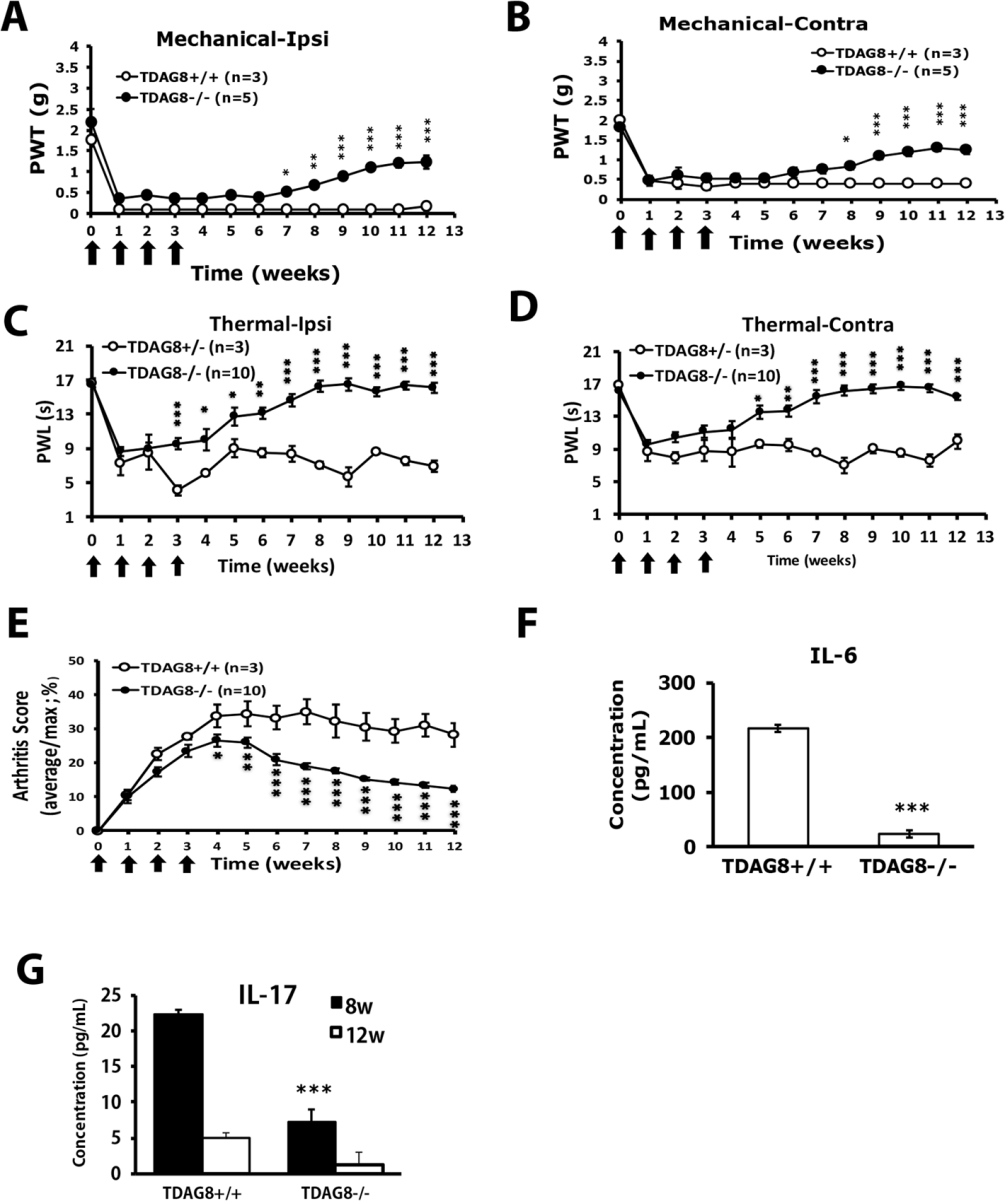
Deletion of TDAG8 gene attenuates mechanical and thermal hyperalgesia and reduces arthritis scores. TDAG8+/+, +/−, −/− mice (8–12 weeks old) were injected with 5 μl of 100% CFA (5 μg) in the right ankle joint (ipsilateral joint) four times at 1-week intervals, then underwent mechanical (**a**, **b**) or thermal (**c**, **d**) behavioral tests. Arrows are CFA treatments. Data are mean ± SEM of total tested mice. *p < 0.05, **p < 0.01, ***p < 0.001, TDAG8^−/−^ (*n* = 5) vs TDAG8^+/+^ (*n* = 3), or TDAG8^−/−^ (*n* = 10) vs TDAG8^+/−^ (*n* = 3). **e** Severity of arthritis presented as arthritis score. *p < 0.05, **p < 0.01, ***p < 0.001, TDAG8^−/−^ (*n* = 10) vs TDAG8^+/+^ (*n* = 3) by two-way ANOVA. **f** IL-6 production at 12 w after the first CFA injection in TDAG8^+/+^ and TDAG8^−/−^ mice. ***p < 0.001, TDAG8^−/−^ (*n* = 3) vs TDAG8^+/+^ (*n* = 3) by Mann-Whitney *U* test. **g** IL-17 production at 8 and 12 weeks after the first CFA injection in TDAG8^+/+^ and TDAG8^−**/**−^ mice. *p < 0.05, TDAG8^−/−^ (*n* = 6) vs TDAG8^+/+^ (*n* = 4) by two-way ANOVA

We previously found that TDAG8 is involved in RA-induced pain and disease progression [19]. Here we used TDAG8 gene-deficient mice to understand how TDAG8 regulates RA. TDAG8 gene deficiency attenuated bilateral mechanical hyperalgesia from week 7 on the ipsilateral side (Fig. 2a) and from week 8 on the contralateral side (Fig. 2b). Bilateral thermal hyperalgesia was attenuated from week 6 at both sides (Fig. 2c, d). Arthritis scores were reduced from week 4 (Fig. 2e). IL-6 production was inhibited at 12 weeks (Fig. 2f) and IL-17 production at 8 weeks in TDAG8-deficient mice (Fig. 2g).

**Fig. 3:**
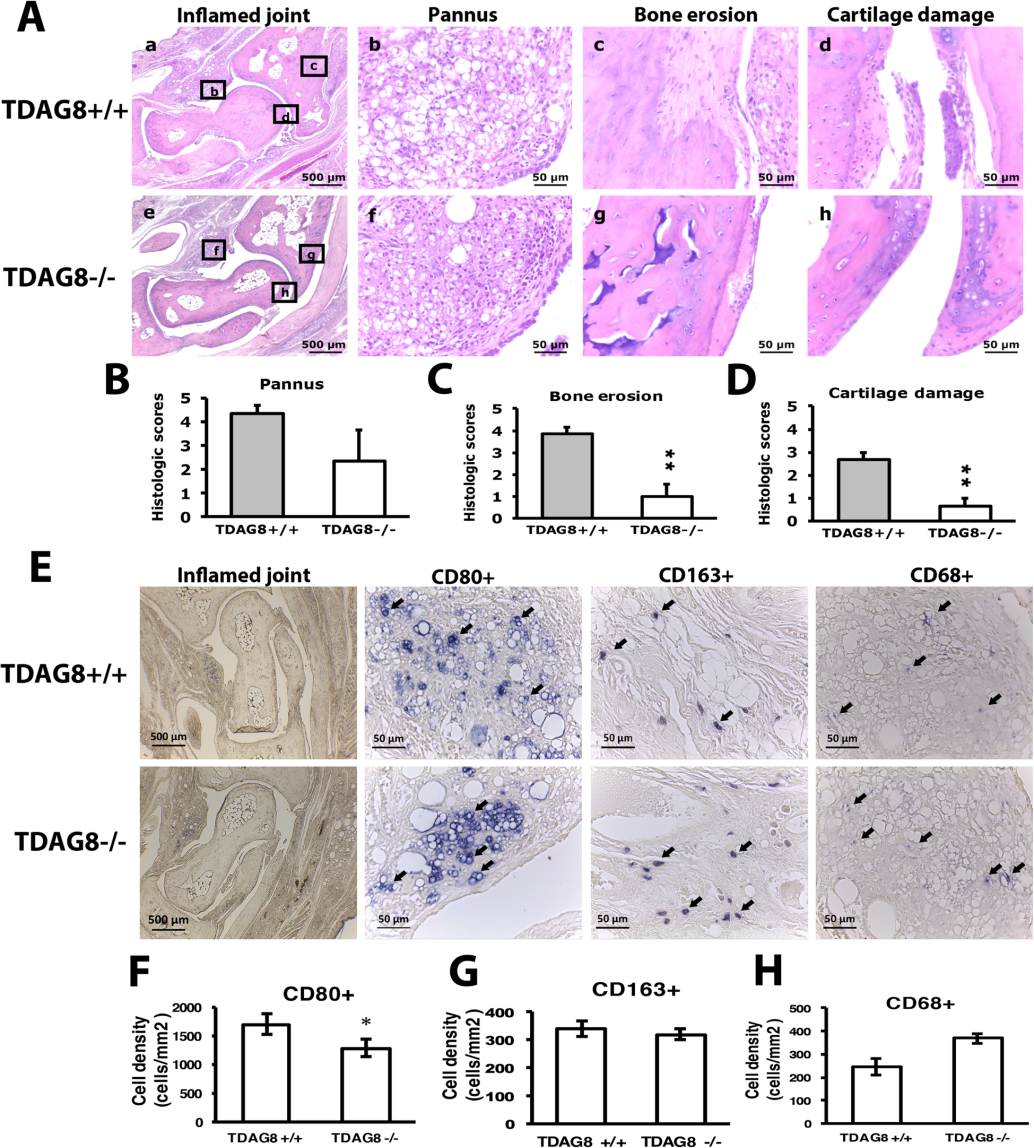
Histology of ipsilateral side joints stained with hematoxylin and eosin or with macrophage markers in TDAG8^+/+^ or TDAG8^−/−^ RA mice. **A**–**D** Histology of joints from TDAG8^+/+^ or TDAG8^−/−^ RA mice at week 12 stained with hematoxylin and eosin. **A** Inflamed joint images (a, e). a for TDAG8^+/+^, e for TDAG8^−/−^; the small letters (b, c, d, f, g, h) in the inflamed joint images represent the regions that were amplified in pannus (b, f), bone erosion (c, g) and cartilage damage (d, h) images. (**B**–**D**) Data are mean ± SEM severity score for pannus (**B**), bone erosion (**C**), and cartilage damage (**D**). **p<0.01 TDAG8^−/−^ vs TDAG8^+/+^ by Mann-Whitney *U* test. (**E**–**H**) Histology of joints from TDAG8^+/+^ or TDAG8^−/−^ RA mice at week 12 stained with CD80 (**F**), CD163 (**G**), or CD68 (**H**) antibodies. Synovial sublining regions are shown in (**E**). Black arrows are labeled cells. The cell density (cells/mm^2^) was presented in histograms representing the mean ± SEM (**F**–**H**). *p < 0.05, TDAG8^−/−^ vs TDAG8^+/+^ by Mann-Whitney *U* test

Consistent with reduced arthritic scores in TDAG8^−/−^ mice, except for pannus, bone destruction and cartilage damage was greatly attenuated in TDAG8^−/−^ mice at week 12 (Fig. 3A–D). We used three macrophage markers to examine macrophage infiltration in joints: CD68^+^ for synovial macrophages and intimal fibroblast-like synoviocytes (FLSs), CD80^+^ for pro-inflammatory M1 macrophages, and CD163^+^ for anti-inflammatory M2 macrophages. We previously found increased CD68^+^ and CD80^+^ cell number with time, corresponding to RA disease progression in mice [19]. Here, TDAG8^−/−^ mice did not show reduced CD68^+^ cell number (Fig. 3E, H), which may reflect lack of significantly reduced pannus. M1 (CD80^+^) macrophage number was significantly decreased at week 12 (Fig. 3E, F), but M2 (CD163^+^) macrophage number remained unchanged (Fig. 3E, G).

**Fig. 4:**
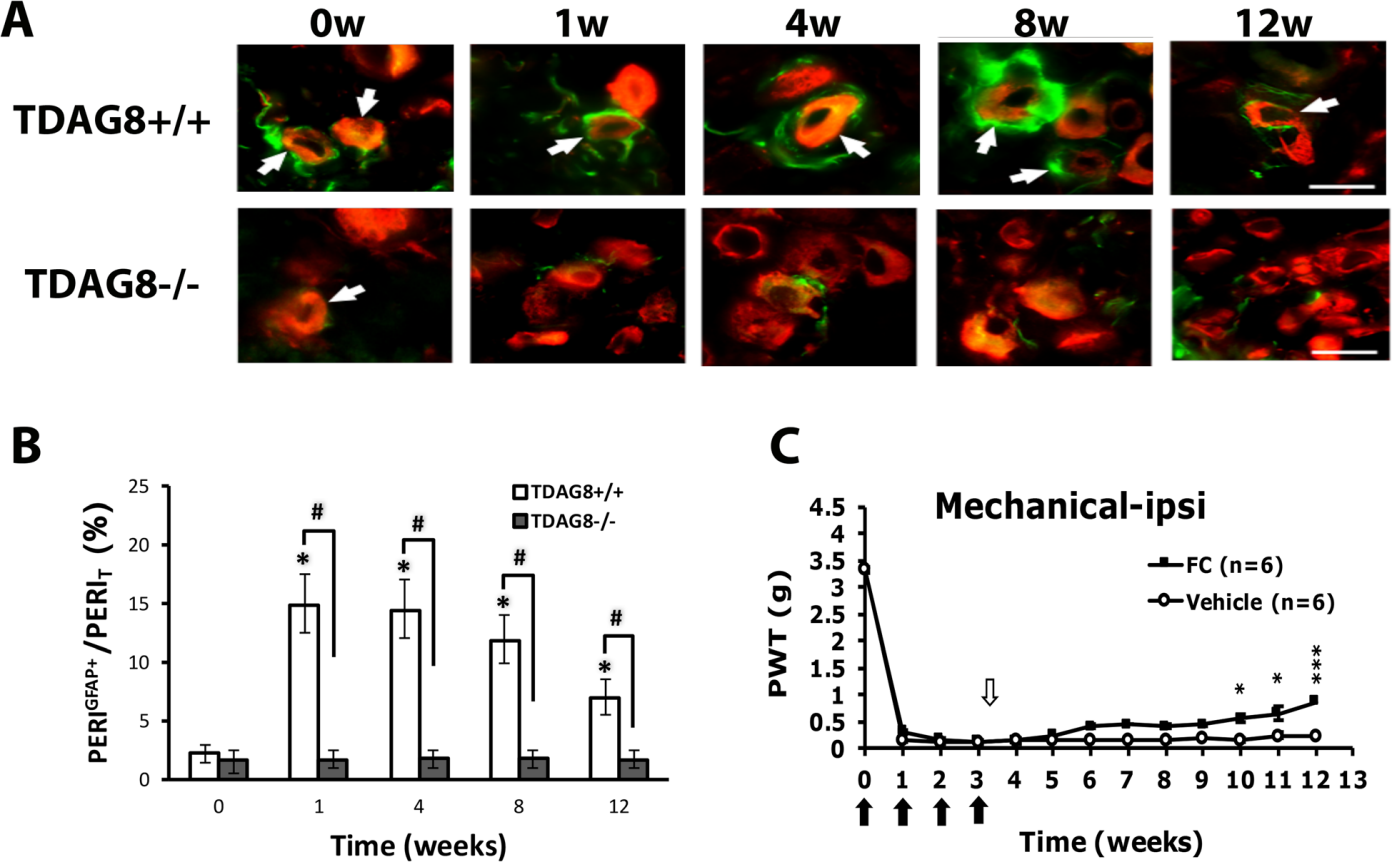
TDAG8 gene deletion reduces the number of satellite glial cells (SGCs) and inhibition of SGCs attenuates chronic phase of RA pain. **a**, **b** DRG from TDAG8^+/+^ or TDAG8^−/−^ RA mice were taken at 0, 1, 4, 8, and 12 weeks after RA induction, then co-immunostained with anti-GFAP and anti-PERI antibodies. **a** Cell images show GFAP- (green) or PERI (red)-positive neurons. Scale bar is 50 μm. **b** Histograms represent the mean±SEM percentage of PERI-positive neurons surrounded by GFAP^+^ cells (PERI^GFAP+^) to total PERI-positive (PERI_T_) neurons. **c** DL-fluorocitric acid (FC) (0.1 nmol) or vehicle was intrathecally injected at week 3 in RA mice, followed by mechanical tests. Data are mean ± SEM of total tested mice (*n* = 6 per group). ***p < 0.001, FC vs vehicle-treated mice by two-way ANOVA

Similar to wild-type RA mice, TDAG8^+/+^ RA mice showed increased number of SGCs, which peaked at the first week (Fig. 4a, b). Nevertheless, the number of SGCs was not increased over time in TDAG8^−/−^ mice (Fig. 4a, b). Intrathecal administration of the SGC inhibitor FC at week 3 after CFA injection attenuated mechanical hyperalgesia from week 10 (Fig. 4c), which suggests that SGCs contribute to the chronic phase of RA-evoked hyperalgesia.

### CCL-2d and LCC-09 relieve RA-induced hyperalgesia

**Fig. 5:**
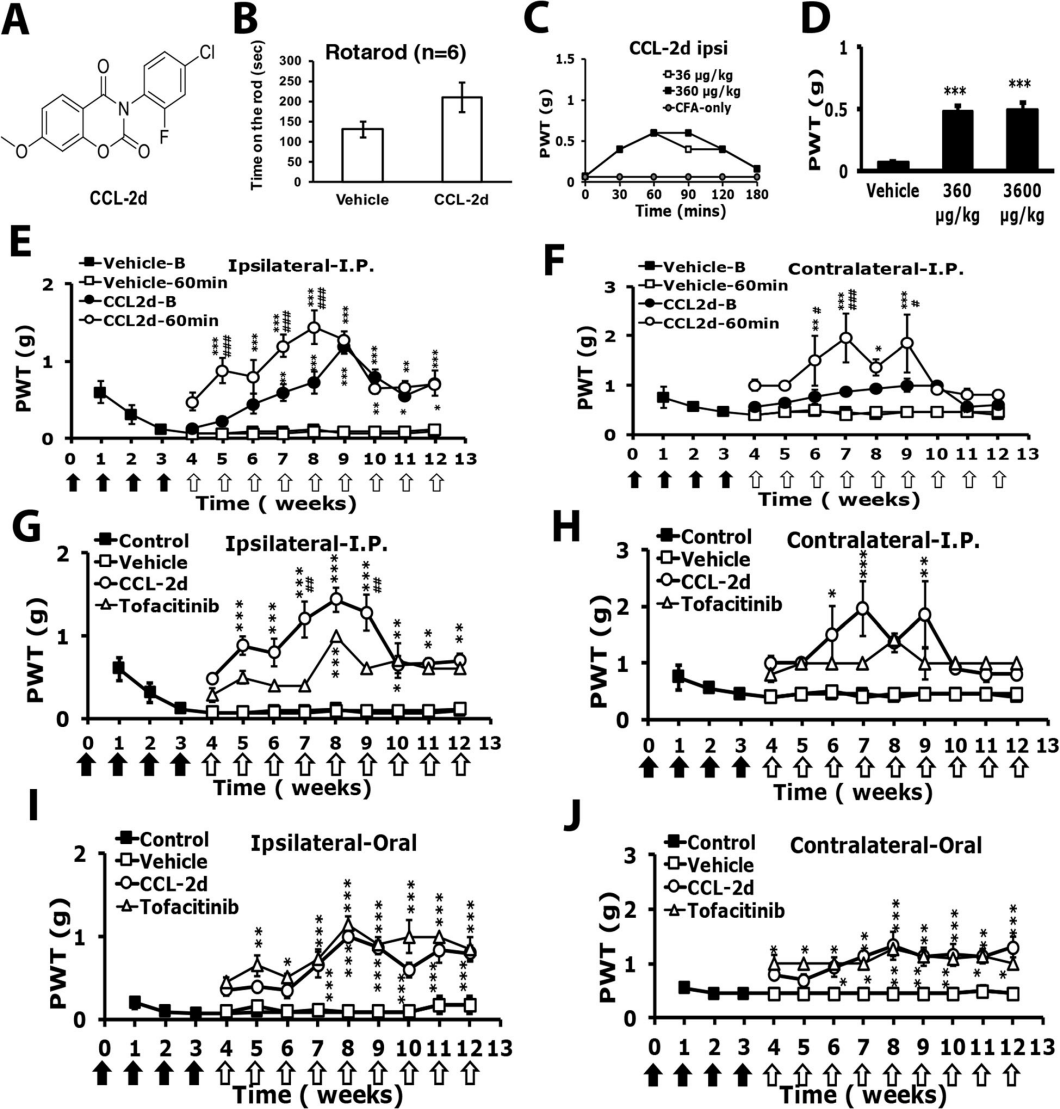
CCL-2d attenuates arthritis-induced mechanical hyperalgesia. **a** CCL-2d chemical structure. **b** Mice (*n* = 6) were intraperitoneally injected with CCL-2d (360 μg/kg), followed by a rota-rod test. **c**, **d** Mice were treated as in Fig. 1. After 4 weeks, mice were intraperitoneally injected with doses of CCL-2d (**a**, 360, 3600 μg/kg), then underwent mechanical behavioral tests with von-Frey filaments. C represents time courses (*n* = 1 for each group). **d** represents PWT at 60 min (*n* = 6). ***p < 0.001 by one-way ANOVA. **e**–**j** For long-term treatments, 360 μg/kg CCL-2d or 3 mg/kg tofacitinib was intraperitoneally (**e**–**h**) or orally (**i**, **j**) administered weekly for 9 consecutive weeks. Mechanical tests were performed before (**b**) or after (60 min) compound injection every week from week 4. Data are mean ± SEM of total tested mice. *p < 0.5, **p < 0.01, ***p < 0.001, vehicle- (*n* = 6) vs CCL-2d-treated (*n* = 6) before (**b**) or at 60 min; #*p* < 0.5, ##*p* < 0.01, ###*p* < 0.001 CCL-2d-treated at 60 min vs before by two-way ANOVA. Black arrows indicate CFA treatments and white arrows are CCL-2d treatments

**Fig. 6:**
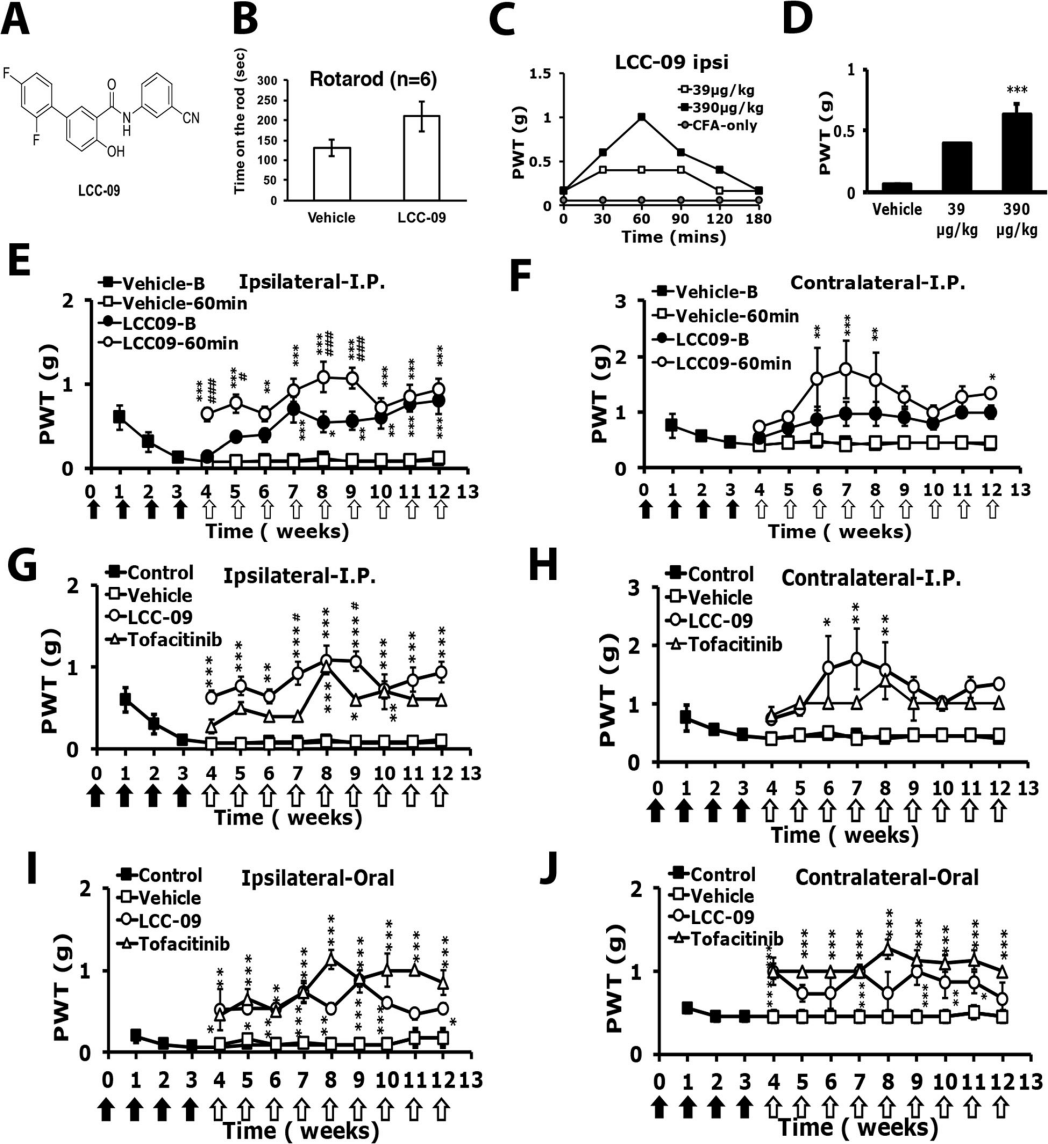
LCC-09 attenuates arthritis-induced mechanical hyperalgesia. **a** LCC-09 chemical structure. **b** Mice (*n* = 6) were intraperitoneally injected with LCC-09 (390 μg/kg), followed by a rota-rod test. **c**, **d** Mice were treated as in Fig. 1. After 4 weeks, mice were intraperitoneally injected once with doses of LCC-09 (**a**, 39, 390 μg/kg), then underwent mechanical behavioral tests with von-Frey filaments. **c** Time courses (*n* = 1 for each group). **d** PWT at 60 min (*n* = 6). ***p < 0.001 by one-way ANOVA. **e**–**j** For long-term treatments, 390 μg/kg LCC-09 or 3 mg/kg tofacitinib was intraperitoneally (**e**–**h**) or orally (**i**, **j**) administered weekly for 9 consecutive weeks. Mechanical tests were performed before (**b**) or after (60 min) compound injection every week from week 4. Data are mean ± SEM of total tested mice (*n* = 6 per group). *p < 0.5, **p < 0.01, ***p < 0.001, vehicle- (*n* = 6) vs LCC-09-treated (*n* = 6) before or at 60 min; #*p* < 0.5, ##*p* < 0.01, ###*p* < 0.001 LCC-09-treated at 60 min vs before by two-way ANOVA. Black arrows are CFA treatments and white arrows are LCC-09 treatments

To further confirm the TDAG8 involvement in RA disease and pain, we tested the previously developed small salicylanilide derivatives CCL-2d (Fig. 5a) and LCC-09 (Fig. 6a) [20, 21] in RA mice. After CFA injection for 4 weeks, mice were intraperitoneally (i.p.) injected once with the compounds at different doses, then underwent a rota-rod test or a mechanical behavioral test. The dosages of CCL-2d were determined by in vitro cell-based and in vivo animal experiments (data not shown). The rotarod results showed no differences between vehicle-treated and CCL-2d-treated mice (Fig. 5b). One-time administration of CCL2-d reduced mechanical hyperalgesia from 30 min, peaked at 60 min and declined at 180 min (Fig. 5c). Doses of 360 and 3600 μg/kg CCL-2d attenuated mechanical hyperalgesia at 60 min (Fig. 5d). CCL-2d 360 μg/kg injected weekly for 9 consecutive weeks effectively reduced the mechanical hyperalgesia in both ipsilateral and contralateral sides, and the analgesic effects improved up to week 9 (Fig. 5e, f), then decreased. The CCL-2d–induced analgesic effect seemed cumulative because the paw withdrawal threshold (PWT) before CCL-2d injection (B) was increased from 6 weeks. As compared with tofacitinib (commercial RA drug, 3 mg/kg), CCL-2d (360 μg/kg) had a greater analgesic effect from weeks 5 to 9 on the ipsilateral side (Fig. 5g, h). Oral administration of CCL-2d also attenuated the mechanical hyperalgesia (Fig. 5i, j), and the analgesic effect of oral administration was similar to that of tofacitinib.

Similar to CCL-2d, no difference between vehicle-treated and LCC-09-treated mice was found in rota-rod tests (Fig. 6b). One-time administration of LCC-09 (390 μg/kg) reduced mechanical hyperalgesia from 30 min, peaked at 60 min and then declined (Fig. 6c). Injection of LCC-09 at 39 or 390 μg/kg reduced mechanical hyperalgesia at 60 min (Fig. 6d). LCC-09 390 μg/kg injected weekly for 9 consecutive weeks effectively attenuated the mechanical hyperalgesia on both sides and the analgesic effects improved up to week 12 (Fig. 6e, f). The analgesic effect of LCC-09 was similar to that of tofacitinib (3 mg/kg) (Fig. 6g, h). Similar results were found with oral administration of LCC-09 (Fig. 6i, j).

### CCL-2d and LCC-09 reduce synovial inflammation, bone erosion, and macrophage infiltration

**Fig. 7:**
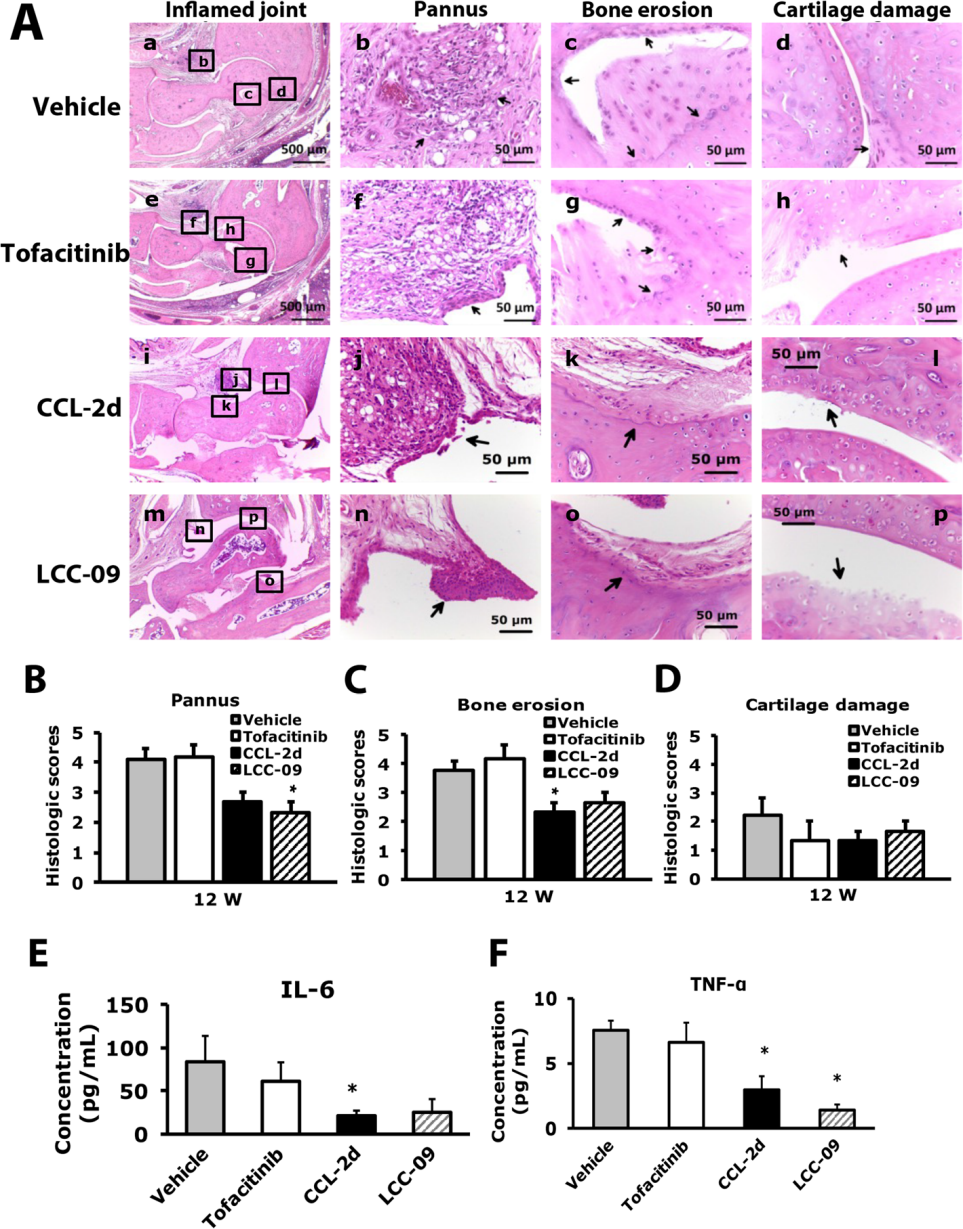
Histology of ipsilateral side joints in compound-treated RA mice. Histology of joints from vehicle- or compound-treated mice at week 12 stained with hematoxylin and eosin. **A** Inflamed joint images (a, e, i m). a for vehicle-treated, e for tofacitinib-treated, i for CCL-2d-treated, m for LCC-09-treated; the small letters (b, c, d, f, g, h, j, k, l, n, o, p) in the inflamed joint images represent the regions that were amplified in pannus (b, f, j, n), bone erosion (c, g, k, o), and cartilage damage (d, h, l, p) images. **B**–**D** Data are mean ± SEM severity score for histological scores for pannus (**B**), bone erosion (**C**), and cartilage damage (**D**). *p < 0.05 compound- vs vehicle-treated group by one-way ANOVA. **E** Serum level of IL-6 *p < 0.05, compound-treated vs vehicle-treated group by one-way ANOVA. **F** Serum level of TNF-α. Data are mean ± SEM of total tested mice (*n* ≥ 6 per group). *p < 0.05, compound-treated vs vehicle-treated group by one-way ANOVA

Histopathological examination of the ankle joints of CCL-2d–, LCC-09–, or tofacitinib-treated mice at 12 weeks revealed that CCL-2d or LCC-09 but not tofacitinib reduced pannus and bone erosion induced by repeated CFA injection (Fig. 7A–D). Pannus was significantly reduced with LCC-09 (Fig. 7B), and bone erosion was significantly decreased with CCL-2d (Fig. 7C). CCL-2d treatment also decreased serum IL-6 level at 12 weeks (Fig. 7E), and both treatments reduced serum TNF-α level at 12 weeks (Fig. 7F). Tofacitinib did not reduce the serum level of IL-6 or TNF-α (Fig. 7E, F).

**Fig. 8:**
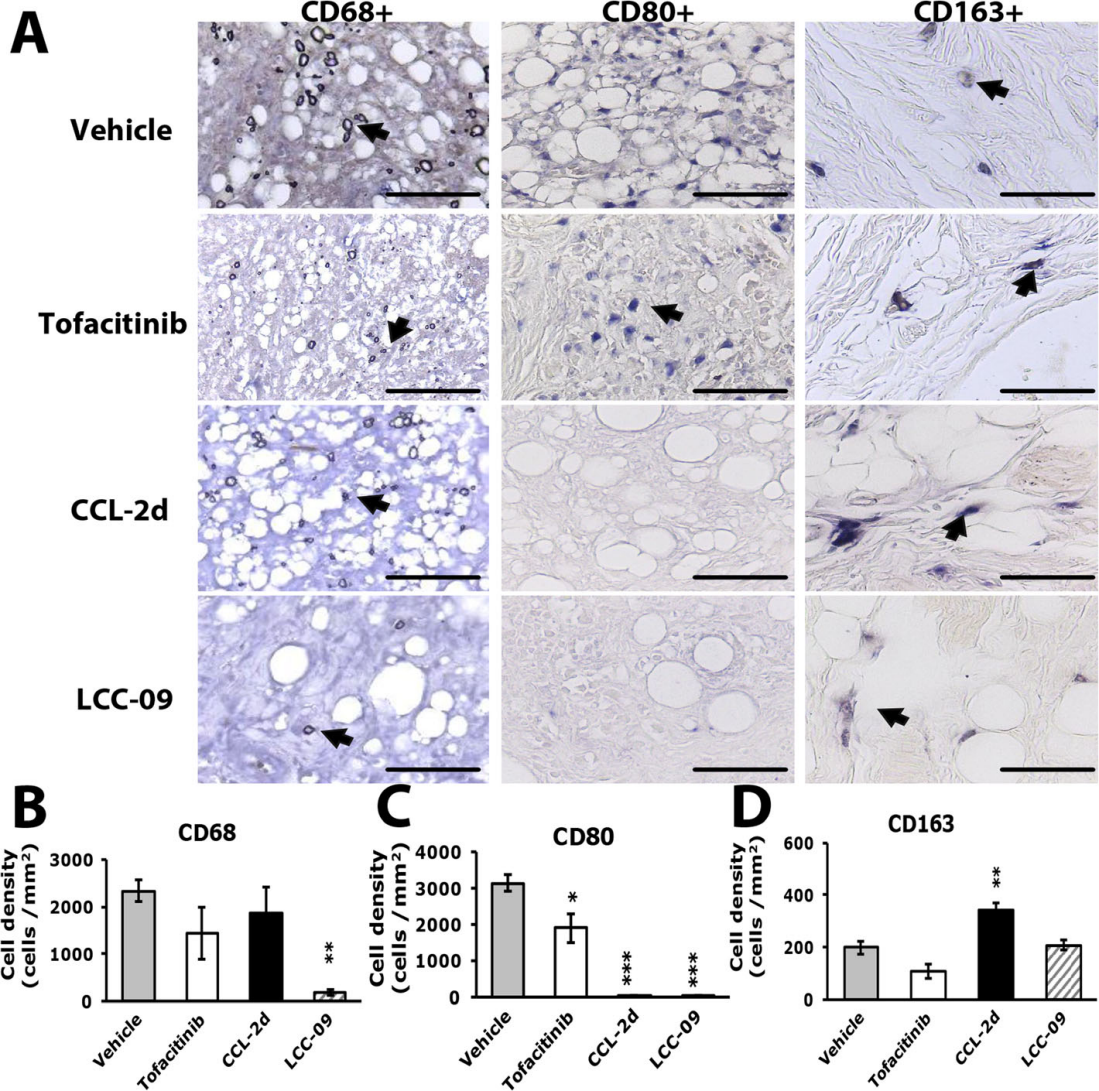
Histology of ipsilateral side joints stained with macrophage markers in compound-treated RA mice. Histology of joints from vehicle- or compound-treated mice at week 12 incubated with CD68 (**b**), CD80 (**c**), or CD163 (**d**) antibodies. Synovial sublining regions are shown in **a**. Magnification of × 40. The cell density (cells/mm^2^) was calculated and presented in histograms representing the mean ± SEM (**b**, **c**, **d**). *p < 0.05, **p < 0.01 compound- vs vehicle-treated group by one-way ANOVA. Yellow arrows are labeled cells

CCL-2d and LCC-09 treatment had distinct effects on macrophages and FLSs. Only LCC-09 significantly reduced CD68^+^ cell number (Fig. 8a, b), which was consistent with reduced pannus (Fig. 8b). However, both LCC-09 and CCL-2d decreased CD80^+^ cell number, and CCL-2d increased CD163^+^ cell number (Fig. 8a, c, d). CD163^+^ macrophages have an anti-inflammatory role, which could explain why CCL-2d treatment did not significantly reduce pannus but decreased bone-erosion and serum IL-6 levels. Although tofacitinib reduced CD80^+^ cell number 33%, it did not reduce the CD68^+^ cell number, which could explain the lack of reduction in pannus with tofacitinib (Fig. 8a–c).

### CCL-2d and LCC-09 suppress TDAG8 gene expression and SGC number

**Fig. 9:**
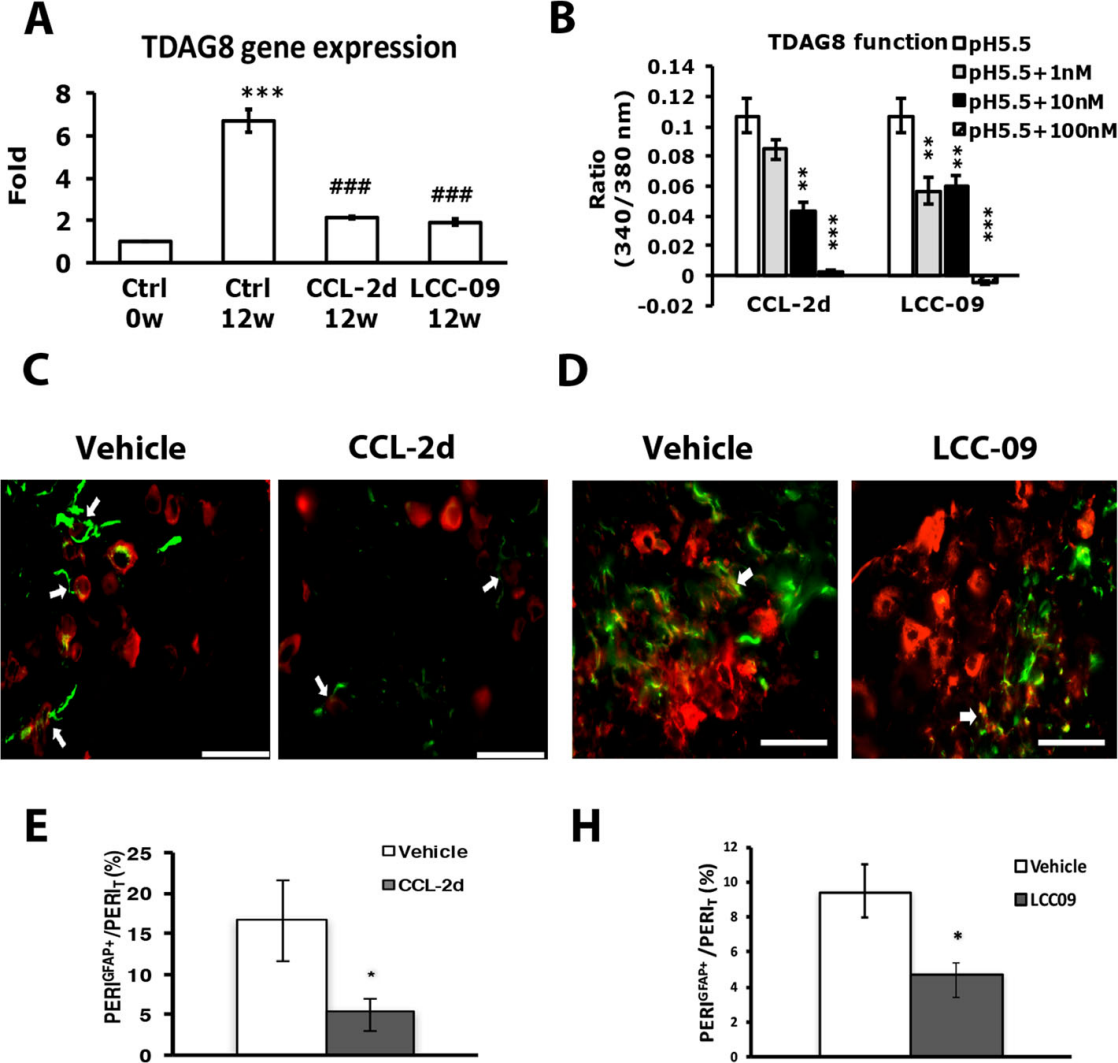
CCL-2d or LCC-09 suppresses TDAG8 expression and function and reduces the number of SGCs in DRG. **a** DRG from control (Ctrl) or compound-treated mice were taken at 0 and 12 weeks after RA induction, then underwent qRT-PCR to measure TDAG8 mRNA expression. Data are mean ± SEM. ***p < 0.001 Ctrl 12 weeks vs Ctrl 0 weeks; ##*p* < 0.01, ###*p* < 0.001, compound-treated 12 weeks vs Ctrl 12 weeks by one-way ANOVA. **b** HEK293 cells were transfected with TDAG8, then incubated with 0.1, 1, and 10 μM CCL-2d or LCC-09, followed by acid (pH 5.5) stimulation. Calcium signals were detected. Data are mean±SEM peak values of signals. *n* = 20–47 cells. **c**–**f** DRG from vehicle- (**c**–**f**), CCL-2d- (**d**), or LCC-09 (**e**)-treated mice were taken at 12 weeks, then co-immunostained with anti-GFAP and anti-PERI antibodies. Cell images (**c**, **d**) showing GFAP- (green) and PERI (red)-positive neurons. Scale bar is 50 μm. (**e**, **f**) Histograms represent the mean ± SEM percentage of PERI-positive neurons surrounded by GFAP^+^ cells (PERI^GFAP+^) to total PERI-positive (PERI_T_) neurons. *p < 0.05 by the *z* test

To understand the mechanism of the analgesic action of CCL-2d or LCC-09, we used DRG from RA mice with or without CCL-2d or LCC-09 treatment and examined TDAG8 gene expression. TDAG8 mRNA expression was increased at 12 weeks in RA mice, which is consistent with our previous study [19]. Both CCL-2d and LCC-09 reduced this increase in expression to the basal level (Fig. 9a). Because both compounds inhibited TDAG8 gene expression, we then examined whether CCL-2d or LCC-09 could inhibit TDAG8 function. Since TDAG8 activation induces cAMP accumulation and intracellular calcium increase [23], we examined TDAG8 function using calcium imaging to detect intracellular calcium increase. TDAG8 was transfected into HEK293 cells. Transfected cells were treated with 1, 10, and 100 nM CCL-2d or LCC-09. CCL-2d 10 nM partially inhibited acid-induced signals in TDAG8-expressing cells, but 100 nM completely inhibited TDAG8-mediated signaling (Fig. 9b). LCC-09 100 nM also inhibited TDAG8-mediated signaling. Hence, CCL-2d and LCC-09 may both inhibit TDAG8 gene expression and function.

We then examined whether CCL-2d or LCC-09 could suppress TDAG8 expression and function to control SGC number. CCL-2d and LCC-09 treatment attenuated the increased number of SGCs at week 12 (Fig. 9c–f), which suggests that CCL2d and LCC-09 could act on TDAG8 to inhibit SGC activation, further regulating bilateral hyperalgesia.

## Discussion

We previously found TDAG8 involved in RA disease progression and associated pain [19]. Here, we demonstrated that RA development increased the number of SGCs, and inhibition of glial activation blocked the chronic phase of RA-associated pain. TDAG8 gene deletion inhibited SGC activation and attenuated the chronic phase of RA-associated pain. Thus, TDAG8 may modulate activation of SGCs to attenuate the chronic phase of RA-associated pain. In addition, TDAG8 deletion reduced the number of pro-inflammatory M1 macrophages, which may explain the decrease in arthritis scores and associated pain in TDAG8-deficient mice. We examined the effects of two salicylanilide derivatives, CCL-2d and LCC-09, which were originally designed to inhibit receptor activator of NF-κB ligand (RANKL)-induced osteoclastogenesis [20, 21] in RA disease progression and associated pain. Both compounds attenuated RA progression and associated pain. These two compounds inhibited TDAG8 gene expression in RA mice and blocked TDAG8 signalling, thereby reducing the number of SGCs and pro-inflammatory M1 macrophages. Thus, TDAG8 may control the number of SGCs and proinflammatory macrophages to modulate RA disease severity and associated pain.

TDAG8 gene deletion greatly attenuated chronic hyperalgesia (> 7 weeks) but less effectively acute hyperalgesia in our mouse model. TDAG8 may regulate different components contributing to the early and late RA pain stages. The acute phase of pain could be largely attributed to acute joint inflammation because of an increase in synovial macrophage number. TDAG8 deficiency reduced the number of pro-inflammatory M1 macrophages (CD80^+^ cells) but not synovial macrophages (CD68^+^ cells). Therefore, the acute hyperalgesia was less attenuated in TDAG8^−/−^ mice, which may explain why arthritis scores were not reduced in the early stage of RA in TDAG8^−/−^ mice. The hypoxia condition may favor M1 polarization [12]. Given that TDAG8 is expressed in macrophages [27–29] and TDAG8 responds to acid [30, 31], mice lacking the TDAG8 gene may have reduced macrophage responsiveness to the hypoxia condition, which would not favor M1 polarization.

In the chronic phase, RA progression mainly depends on the increase in M1 macrophage number and cytokine levels (IL-17 and IL-6). Given that both IL-6 and IL-17 are involved in arthritis-induced hyperalgesia [32–34] and administration of a macrophage blocker reduced hyperalgesia [35], the reduced M1 macrophage number and IL-6 and IL-17 levels we showed may have synergistically contributed to a decline in chronic hyperalgesia and arthritis score. Moreover, neuron–glia interactions play a role in the chronic phase. Consistent with previous studies in monoarthritis or osteoarthritis [13, 14], our RA mice showed increased number of SGCs. With TDAG8 deficiency, the increased number of SGCs was completely inhibited. Administration of an SGC inhibitor at week 3 attenuated the chronic hyperalgesia (from 6 weeks). Reduced chronic hyperalgesia caused by TDAG8 deletion could also be attributed to an inhibition of the increased SGC number. This suggestion could explain why hyperalgesia was greatly attenuated in the late phase in TDAG8^−/−^ mice. SGCs can be activated by substance P or calcitonin gene-related peptide (CGRP) [36] or by IL-17 [15]. Given that TDAG8 gene knockdown in a peripheral nerve reduces neuron activity [23], TDAG8 deficiency may reduce neuron activity and release of substance P or CGRP, thereby blocking SGC activation. TDAG8 gene-deficient mice show reduced number of Th17 cells and secretion of IL-17A [18]. Alternatively, TDAG8 deficiency could decrease IL-17 levels to directly attenuate RA-induced hyperalgesia or indirectly affect SGC activation thereby reducing hyperalgesia. TDAG8^−/−^ mice showed reduced IL-17 level from week 8, which corresponded to attenuated hyperalgesia in the chronic phase.

A previous study of TDAG8–deficient mice found that TDAG8 deficiency promotes arthritis using anti-collagen antibody/lipopolysaccharide model [37], suggesting that TDAG8 is a negative regulator for RA. However, recent studies in human patients found that spondyloarthritis patients are associated with genetic variants of TDAG8 locus [16], and their Th17 cells show high expression of TDAG8 gene [17]. TDAG8 gene-deficient mice show reduced number of Th17 cells and secretion of IL-17A [18]. These results suggest that TDAG8 is a positive regulator for RA progression, which are consistent with our RA animal results.

Similar to results from TDAG8-deficient mice, consecutive administration of the salicylanilide derivative CCL-2d or LCC-09 suppressed TDAG8 expression and function, further reducing the bilateral mechanical hyperalgesia induced by arthritis. Both CCL-2d and LCC-09 treatments also reduced the number of SGCs and M1 macrophages. These two compounds likely act on TDAG8 to reduce the proliferation of SGCs and M1 macrophage polarization, thereby affecting bilateral hyperalgesia.

Low doses of CCL-2d (360 μg/kg) or LCC-09 (390 μg/kg) had similar analgesic effects as 3 mg/kg tofacitinib injection, which suggests that CCL-2d or LCC-09 could be more effective analgesics than tofacitinib. The analgesic effect of CCL-2d or LCC-09 was transient (lasted 90–120 min) for a single-dose injection, but consecutive injection (once per week) seemed to have synergistic effects in later weeks. Mechanical hyperalgesia before each injection (CCL-2d 0 min or LCC-09 0 min) was gradually attenuated after week 5 (after the first injection). The reason for the synergistic effects is unclear. Given that both compounds inhibit TDAG8 expression and TDAG8-deficient mice also showed gradually attenuated mechanical hyperalgesia from week 7, the synergistic effects of CCL-2d or LCC-09 on hyperalgesia could be attributed to long-term suppression of TDAG8 gene expression and function.

Although TDAG8 gene deletion significantly reduced RA pain, arthritis scores, bone erosion and cartilage damage, it only slightly reduced pannus. Less reduction in synovial inflammation could be due to distinct effects on macrophages and synovial fibroblasts that are predominantly expressed in the synovial lining of the joint and play many roles in synovial inflammation [38, 39]. Both synovial macrophages and intimal fibroblast-like synoviocytes are positive for CD68, whereas CD80 recognizes macrophages in synovial sublinings, bone, and other joint areas. Given that TDAG8 deficiency reduced the increased number of CD80^+^ macrophages with no inhibition of CD68^+^ macrophages, TDAG8 may regulate macrophages but not synoviocytes, which explains the slight reduction of pannus. Similarly, CCL-2d greatly decreased CD80^+^ but not CD68^+^ cell number, for only a slight reduction in pannus. In contrast, LCC-09 significantly decreased CD68^+^ and CD80^+^ cell number, which agrees with the reduced pannus.

The presence of osteoclasts, a form of macrophages in bone, seems essential for RA-induced bone erosion because articular bone remains well preserved in the absence of osteoclasts despite overexpression of proinflammatory cytokines [40]. TDAG8 deficiency reduced CD80^+^ cell number, so it probably also decreased osteoclasts to prevent bone erosion. Acidosis in joints induces chondrocyte apoptosis [41–43], which may explain the prevention of cartilage damage in TDAG8-deficient mice.

In our previous study of TDAG8, gene expression suppressed in peripheral nerves of mice, only the initial phase of RA-induced pain was reduced with TDAG8 knockdown [19]. Nervertheless, in the current study, both the initial and chronic phases of RA pain were reduced in TDAG8-deficient mice. Given that suppression of TDAG8 reduced CD68^+^ and CD80^+^ cell number [19] but TDAG8 gene deficiency decreased only CD80^+^ cell number, CD68^+^ cells could be the major factor for the initial development of RA pain rather than the chronic phase, whereas CD80^+^ could contribute to both the initial and chronic phase of RA pain. The finding also explains the attenuated initial arthritis scores in TDAG8-knockdown mice but not TDAG8 gene-deficient mice. Alternatively, we have previously found that ASIC3, TRPV1, and TDAG8 are involved in RA progression and associated pain [19]. Therefore, we have examined ASIC3 and TRPV1 gene expression in TDAG8 knockout mice without RA induction and found no significant change in ASIC3 and TRPV1 gene expression. It suggests that no ASIC3 and TRPV1 influence on TDAG8 knockout mice before RA induction, although it cannot be excluded a possibility that TDAG8 knockout mice have declined expression levels of ASIC3 and TRPV1 genes after RA induction. In both ASIC3 and TRPV1 knockout mice with attenuated chronic hyperalgesia, expression levels of all three genes were eliminated or at low levels at 12 weeks after RA induction. It is likely that high expression levels of ASIC3, TRPV1, and TDAG8 at 12 weeks after RA induction are essential for maintenance of chronic hyperalgesia.

Accordingly, TDAG8 gene expression in peripheral nerves could be critical to regulate CD68^+^ and CD80^+^ cell number, thus contributing to the initial development of RA and associated pain, and TDAG8-mediated satellite glial activation and increased IL-6 and IL-17 levels could be essential for maintenance of RA pain.

## Conclusions

This study demonstrated that TDAG8 gene deficiency reduced SGC number, and SGC inhibition attenuated the chronic phase of RA pain, which suggests that TDAG8 deficiency relieved the late phase of RA pain by regulating SGCs in part. Moreover, reduced M1 macrophage number but not synovial macrophage number in TDAG8-deficient mice may explain the less attenuation of the acute phase of RA disease activity and associated pain. These results reveal how the acute and chronic phases of RA pain are regulated by TDAG8 through M1 macrophages and SGCs. M1 macrophages are critical for the development and maintenance of RA disease and pain, but statellite glial activation is also required for the chronic phase of RA pain. These findings provide further insights for developing threapeutic treatments for RA disease and associated pain at different stages.

## Data Availability

All raw data used in this manuscript are available from the corresponding author on reasonable request.

## Abbreviations

RA: Rheumatoid arthritis
TDAG8: T cell death-associated gene 8
CFA: Complete Freund’s adjuvant
SGC: Satellite glial cells
DRG: Dorsal root ganglia
FC: DL-fluorocitric acid
TNF: Tumor necrosis factor
IL-6: Interleukin 6
IL-17: Interleukin 17
Th17: T-helper 17
H&E: Hematoxylin and eosin:
HEK293T: Human embryonic kidney, adenovirus type 5-transformed 293 cells
PERI: Peripherin
GFAP: Glial fibrillary acidic protein
FLS: Fibroblast-like synoviocytes
RANKL: Receptor activator of NF-κB ligand
CGRP: Calcitonin gene-related peptide
